# Automated Detection of Aberrant Episodes in Epileptic Conditions: Leveraging EEG and Machine Learning Algorithms

**DOI:** 10.3390/bioengineering12040355

**Published:** 2025-03-29

**Authors:** Uddipan Hazarika, Bidyut Bikash Borah, Soumik Roy, Manob Jyoti Saikia

**Affiliations:** 1 Department of Electronics and Communication Engineering, Tezpur University, Sonitpur 784028, Assam, India; 2 Electrical and Computer Engineering Department, University of Memphis, Memphis, TN 38152, USA; 3 Biomedical Sensors & Systems Lab, University of Memphis, Memphis, TN 38152, USA

**Keywords:** EEG, electroencephalogram, epilepsy, EEG signal processing, Hurst exponent, machine learning, seizure detection

## Abstract

Epilepsy is a neurologic condition characterized by recurring seizures resulting from aberrant brain activity. It is crucial to promptly and precisely detect epileptic seizures to ensure efficient treatment. The gold standard electroencephalography (EEG) accurately records the brain’s electrical activity in real time. The intent of this study is to precisely detect epileptic episodes by leveraging machine learning and deep learning algorithms on EEG inputs. The proposed approach aims to evaluate the feasibility of developing a novel technique that utilizes the Hurst exponent to identify EEG signal properties that could be crucial for classification. The idea posits that the prolonged duration of EEG in epileptic patients and those who are not experiencing seizures can differentiate between the two groups. To achieve this, we analyzed the long-term memory characteristics of EEG by employing time-dependent Hurst analysis. Together, the Hurst exponent and the Daubechies 4 discrete wavelet transformation constitute the basis of this unique feature extraction. We utilize the ANOVA test and random forest regression as feature selection techniques. Our approach creates and evaluates support vector machine, random forest classifier, and long short-term memory network machine learning models to classify seizures using EEG inputs. The highlight of our research approach is that it examines the efficacy of the aforementioned models in classifying seizures utilizing single-channel EEG with minimally handcrafted features. The random forest classifier outperforms other options, with an accuracy of 97% and a sensitivity of 97.20%. Additionally, the proposed model’s capacity to generalize unobserved data is evaluated on the CHB-MIT scalp EEG database, showing remarkable outcomes. Since this framework is computationally efficient, it can be implemented on edge hardware. This strategy can redefine epilepsy diagnoses and hence provide individualized regimens and improve patient outcomes.

## 1. Introduction

Epilepsy is a neurological condition defined by a continual tendency to generate seizures, which are not triggered by an immediate central nervous system insult [1]. Seizures happen due to aberrant electrical impulses in the brain, which momentarily impair normal brain activity and functioning [2]. This can manifest in various ways, from uncontrollable muscle movements to temporary confusion or loss of consciousness [3]. It affects people of all sexes and all ages, with adult males slightly more likely to be affected than women [4]. Depending on the parts of the brain involved and the kind of seizure activity, there are several types of seizures, which are often classified as partial seizures (simple and complex) and generalized seizures (absence, atonic, tonic–clonic, and myoclonic) [5].

The prevalence of epilepsy is estimated to be 5–9 per 1000 population, with a lifetime prevalence of 7.60 per 1000 persons. The incidence rate of epilepsy is estimated to be around 50–60 per 100,000 person-years, indicating that approximately 67.77 per 100,000 persons experience epilepsy annually [1,5]. The incidence rate is higher in low-to-middle-income countries and increases with age, particularly after age 50 [6]. Nearly half a million individuals throughout the globe suffer with epilepsy, making it one of the most common neurological disorders [7]. People with epilepsy may also face social rejection (stigma), mental health problems (psychiatric comorbidity), and significant financial burdens.

Clinical diagnoses vary throughout practitioners due to the subjective nature of electroencephalogram (EEG) interpretation. One major drawback is the possibility of human oversight, such as incorrectly interpreting EEG signals. Research shows that while evaluating the identical EEGs, even experienced neurologists might differ dramatically [8]. Magnetic resonance imaging (MRI) provides great structural information and can detect lesions associated with epilepsy due to its hemodynamic nature [9]; however, it cannot detect functional abnormalities like seizures in real time. As such, it is often used in conjunction with EEG to provide a thorough assessment [10]. Although imaging methods such as functional magnetic resonance imaging (fMRI) and positron emission tomography (PET) offer useful insights, their high cost, restricted availability, lack of portability, and demand that the patient stay still and steady—which is not possible during a seizure—make them unsuitable for regular seizure detection [11].

For the non-invasive measurement of brain electrical activity, an EEG is utilized [12,13]. To collect the electrical impulses generated by neural activity and ionic perturbation across the membrane, electrodes are placed on the scalp. Due to its ability to capture real-time brain activity, EEG is widely used in clinical and research settings for the diagnosis and monitoring of neurological illnesses including epilepsy [14]. EEG has a number of noteworthy benefits when it comes to identifying epileptic seizures and other fleeting neurological occurrences. Its great temporal resolution, which makes it possible to monitor quickly changing brain activity in real time, is its main advantage [15]. This capacity is particularly useful for diagnosing and treating epileptic seizures, which are defined as abrupt and irregular electrical discharges in the brain [16,17]. The affordability and accessibility of EEG are two other noteworthy benefits. Since EEG is less expensive than other neuroimaging methods like MRI and PET, it may be used in a variety of settings, such as outpatient clinics and hospitals [18]. Its affordability, ease of use, straightforward and patient-friendly non-invasive procedure make it more relevant in routine clinical settings, especially in areas with minimal medical resources [17,19].

Waveforms including spikes, sharp waves, and spike-and-wave complexes are examples of epileptiform discharges, which are a sign of epileptic activity. Seizures can be reliably identified by the frequency and presence of certain peculiar patterns or biomarkers in an EEG signal. Since the existence of these unique EEG patterns closely corresponds with the emergence of seizure episodes, they are essential for the diagnosis and ongoing monitoring of epilepsy [18,20]. The EEG frequently displays rhythmic and repeated patterns during a seizure, which are seldom present during regular brain function. These patterns may differ in terms of frequency and amplitude depending on the type of seizure and the specific cortex of the brain affected [17,21]. High-frequency oscillations (HFOs) are commonly seen during seizures and can serve as biomarkers for epileptic activity. HFOs generally occur in the 80–500 Hz range. They are invaluable in determining the epileptogenic zone and the starting point of seizures by examining the EEG [11,22,23]. The development of automated seizure detection algorithms that quickly and accurately identify epileptiform discharges and other seizure-related patterns from electroencephalography data has been made possible by recent advances in machine learning (ML) and signal processing [24,25].

The goal of this research is to propose a framework to rapidly and precisely detect epileptic episodes by using ML algorithms on EEG signals. The identification of epileptic episodes from EEG data is facilitated by the use of feature extraction and feature selection techniques. Together, the Hurst exponent and the Daubechies 4 discrete wavelet transformation (DWT) constitute the basis of the feature extraction technique. The framework employs the analysis of variance (ANOVA) test and random forest regression techniques for the feature selection purpose. The support vector machine (SVM), random forest (RF) classifier, and long short-term memory (LSTM) network are the three ML models that have been developed and tested in this study for the purpose of seizure classification utilizing EEG data.

The principal contributions of this research paradigm are as follows:

- Our research framework investigates the effectiveness of several ML models in classifying epileptic episodes based on single-channel EEG signals using minimally handcrafted features.
- The proposed research inspects the significance of feature extraction and feature selection methods for categorizing EEG signals using ML algorithms. Our study primarily utilizes Hurst exponents and discrete wavelet transform methods for feature engineering. We employed logistic regression to determine the importance of each feature.
- We attempt to systematically assess the created models utilizing k-fold cross-validation (k = 10) with other significant performance metrics. The most appropriate model is under consideration for EEG-based classification, which can aid medical professionals in reliably identifying epileptic episodes from raw EEG signals.
- We are comparing the best model we obtained throughout the study with the state-of-the-art models available to the scientific community. The comparative study proves that our proposed method is superior/equivalent to others in terms of accuracy, as well as its false positive rate, which enables it to obtain high precision compared to others.
- We endeavor to evaluate the generalizability of the proposed model in order to comprehend its performance on unseen data. Additionally, in this investigation, we evaluate the computational efficacy of our framework to determine whether it is feasible to incorporate it into real-world medical hardware devices.

The remaining section of this manuscript addresses the related literature, in-depth flow of the methodology adopted, discussions on the results obtained, and conclusion to address the performance of the proposed work. In Section 2, the performance of various articles related to the classification and detection of episodes in epileptic conditions using EEG and ML techniques and their respective research gaps is studied. Section 3 illustrates the methodological framework of this research, starting from EEG data collection and preprocessing, feature extraction, and selection to the development of ML models for the classification of seizures from EEG data. Section 5 examines the efficacy of the three categorization models, facilitating their assessment, as well as comparing the performance of the proposed study with the existing literature. Lastly, Section 6 addresses the contribution of our research framework and possible dimensions toward which this work may be extended.

## 2. Related Literature

Millions of people throughout the globe are afflicted with epilepsy, which is a neurological illness marked by repeated, unprovoked seizures. There has been a lot of focus on using EEG data to identify epileptic seizures recently, especially with the development of sophisticated ML methods. In order to improve the efficacy and precision of epilepsy detection, this literature review looks at recent developments in the field, focusing on the methodology, datasets, and ML models that were used.

Utilizing ML algorithms, the EEG data may be classified to distinguish between normal patterns and those indicative of epileptic seizures. Shah et al. [26] introduce an innovative method for categorizing epileptic episodes by utilizing a random neural network (RNN) which has undergone training and testing using two publicly accessible datasets: CHB-MIT and BONN, respectively.

Kunekar et al. [27] conduct a comprehensive evaluation of many ML models, such as logistic regression, K-nearest neighbors (KNN), SVM, artificial neural networks (ANNs), and LSTM. They employ the UCI-Epileptic Seizure Dataset to assess the merits and drawbacks of each model. The SVM classifier achieves a validation accuracy of 97.2%, indicating its potential to accurately detect seizures.

Yang et al. [28] introduce an innovative method for categorizing pediatric epilepsy syndromes utilizing a combination of variables extracted from EEG and electrocardiogram (ECG) signals. The primary goal is to evaluate whether the utilization of multimodal physiological signals can enhance the classification accuracy in comparison to employing a single physiological signal. The study used data from the Children’s Hospital at Zhejiang University School of Medicine, specifically examining two prevalent childhood epilepsy syndromes: infantile spasms (commonly referred to as West syndrome) and childhood absence epilepsy (CAE). EEG and ECG data were captured during both seizure (ictal) and non-seizure (interictal) phases.

EEG readings that exhibit symmetry are generally indicative of normal brain function, but asymmetry can be indicative of epileptic activity. Seizures frequently result in abrupt electrical spikes in a single hemisphere of the brain, resulting in asymmetric patterns in the EEG. Yogarajan et al. [29] present a novel approach for improving the automatic seizure detection system by utilizing a deep neural network (DNN) and binary dragonfly algorithm (BDFA). The system utilizes EEG signals to extract features, which are then reduced using BDFA.

Tuncer et al. [30] present a technique for automatically identifying and categorizing seizures from EEG data by employing both conventional and deep learning methods. The research is centered around two classification scenarios: one is a four-class problem that includes different forms of complex partial seizures (CPSZ) and absence seizures (ABSZ), while the other is a two-class problem that aims to differentiate between CPSZ and ABSZ. Preprocessing of EEG data, extraction of features using discrete wavelet transform, and selection of features using Correlation-based Feature Selection (CFS) are carried out. The classification task is accomplished via KNN, SVM, RF, and LSTM algorithms. The approach is applied to 23 participants from the Temple University Hospital EEG dataset.

The objective of the study by Mardini et al. [31] is to improve the identification of epileptic seizures and reduce computational costs. The proposed system utilizes 54-DWT mother wavelets to evaluate EEG data. It employs several ML classifiers such as SVM, KNN, ANN, and Naive Bayes. A total of 14 two-class epilepsy detection combinations are investigated using four ML classifiers. Four classifiers provide comparable outcomes when applied to statistical characteristics created by 54-DWT mother wavelets.

Orosco et al. [32] devise a patient-neutral algorithm for the detection of seizures in scalp EEG signals during their research. The algorithm is designed to enhance the reliability of epilepsy diagnosis through the use of EEG technology by concentrating on generalized features that are consistent across patients and achieved a sensitivity of 87.5%.

Table 1 represents a summary of some of the existing literature and provides insights about their classification techniques, the type of dataset used, the inferences drawn, and their shortcomings or limitations.

### Existing Gaps in the Literature

The majority of the literature on this research problem employs conventional feature extraction methods to obtain time-domain features [26,28,30,31] such as mean amplitude, variance, standard deviation, skewness, kurtosis, etc., as well as frequency-domain features [28,29,30,32] such as power spectral density (PSD), dominant frequency, spectral entropy, individual band power, etc. Additionally, wavelet transforms are employed to obtain time-frequency features [26,29,30,31,32]. The research conducted by Kunekar et al. [27] utilizes deep learning techniques, therefore directly feeding the time-series EEG data into the classifier without employing traditional feature extraction methods. EEG signals demonstrate nonlinear characteristics; hence, employing feature extraction techniques to capture these nonlinear dynamics is recommended. To bridge this research gap, we employed the DWT and Hurst exponent methodologies to extract the time-frequency as well as the nonlinear characteristics inherent in EEG. Additionally, the majority of the research does not investigate the significance of feature selection in the classification of epileptic conditions. However, some of the literature [30,31,32] investigates the significance of feature selection. Therefore, in order to resolve this discrepancy, we implemented feature selection strategies that included the ANOVA test and random forest regression.

Table 1 presents the limitations noticed in the relevant literature. The research conducted by Shah et al. [26] has a notable limitation in that it combines multi-channel (from CHB-MIT) EEG with single-channel (University of Bonn) EEG data from multiple patients, which could lead to potential information leakage between the training and testing datasets. The presence of this overlap may lead to an inflated assessment of the model’s ability to generalize. The study conducted by Kunekar et al. [27] demonstrates a high computational cost, potentially limiting its applicability in resource-constrained environments. The analysis conducted by Yang et al. [28] reveals a notable limitation in the form of a restricted sample size within the dataset from the Children’s Hospital at Zhejiang University School of Medicine. Furthermore, there was no mention of the evaluation regarding the computing efficiency of the method. The study conducted by Yogarajan et al. [29] demonstrates significant computational costs, and it lacks information regarding the size and diversity of the dataset utilized. The framework put forth by Tuncer et al. [30] exhibits notable limitations, including high computational demands and a lack of variability in the dataset utilized. The research proposed by Mardini et al. [31] presents a significant drawback due to its substantial computational requirements, which may impede its implementation in clinical settings where timely decision-making is essential. Without patient-specific calibration, the algorithm put forth by Orosco et al. [32] could misclassify normal EEG readings as seizures, leading to a higher rate of false positives.

To bridge the aforementioned limitations in the existing literature, we are compelled to propose a framework capable of accurately and rapidly detecting epileptic episodes from EEG data while being computationally efficient for implementation in real-world scenarios with limited resources. One of the primary goals of our research framework is to enhance the generalizability of unseen data. The issue of errorneous classification, as identified in the work of Orosco et al. [32], serves as an incentive for us to develop our research in a manner that achieves a low false positive rate, which is crucial for the medical diagnosis of epilepsy.

## 3. Materials and Methods

Epilepsy is a collection of neurological disorders distinguished by recurring epileptic seizures. These seizures can induce intense convulsions, potentially resulting in significant self-inflicted harm. Fortunately, epilepsy is a treatable condition, with 70% of cases being manageable through early detection and medication [33,34]. Over time, researchers have developed numerous diagnostic methods for epilepsy to facilitate early detection. Out of these methods, the technique involving ML with EEG signals as input for the early or timely detection of epilepsy is widely appreciated. However, the primary challenge of this technique is that the visual inspection process for distinguishing EEGs is both time-consuming and expensive.

**Figure 1:**
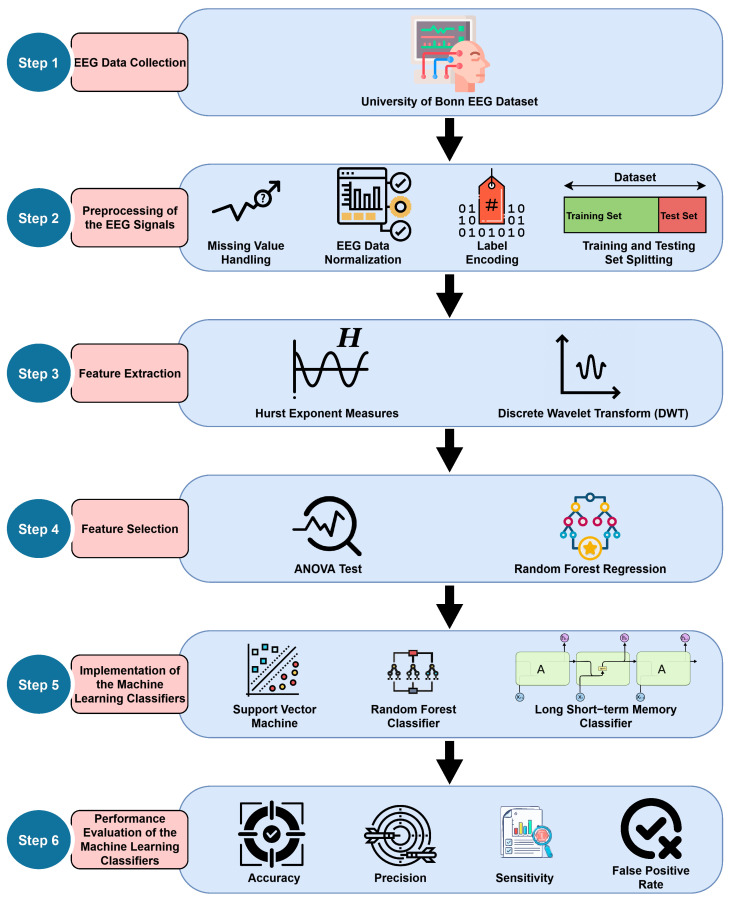
A block diagram flow representation of the proposed methodology.

The objective of this research is to achieve rapid and precise detection of epileptic seizures by employing ML and deep learning techniques on EEG brain data signals. This study will contribute to enhancing the treatment of epilepsy by leveraging engineering principles. The detection of epileptic episodes from EEG signals involves the application of Hurst exponent analysis and discrete wavelet transform for extracting meaningful features. Following these modifications, we use the extracted data as input for SVM, RF, and LSTM algorithms. Based on logical reasoning, there is only one possible outcome for the identification of an epileptic seizure. If an individual experiences epileptic seizures, the resulting output is logically 1. Otherwise, it is a logical 0. Figure 1 shows a flow representation of the proposed methodology.

### 3.1. Data Collection and Preprocessing

#### 3.1.1. Description of the EEG Dataset

Our research uses the University of Bonn EEG dataset [35,36], which consists of EEG recordings from individuals both with and without epileptic seizures. The University of Bonn EEG dataset is widely acknowledged in the field of epilepsy research and ML, namely for its utility in the advancement of seizure detection algorithms. This dataset contains five categories (labeled A to E) representing distinct types of EEG activity, with a specific emphasis on differentiating seizure activity from non-seizure activity. Each of the subsets includes 100 segments of a single-channel EEG signal. Each data segment comprises 23.6 s of EEG signals recorded at a sampling rate of 173.61 Hz. Table 2 and Table 3 describe the dataset with collection phase and structure, and attributes, respectively.

#### 3.1.2. Data Preprocessing

Data preprocessing is a vital stage in the data analysis mechanism, especially when dealing with intricate datasets like EEG recordings. Data cleansing is a crucial operation in this step, since it entails removing superfluous columns and optimizing the dataset for analysis. When processing signals, a bandpass filter with a pass-range of 0.53–40 Hz is applied to eliminate unwanted frequencies and noise. The missing value handling approach is employed to identify Not-a-Numbers (NaNs), zeros, constant values, and flat segments exhibiting no fluctuation.

The data were subsequently normalized to mitigate numerical instability and enhance consistency. In order to ensure that each feature contributes equally to the analysis and to improve performance, normalization is a critical prerequisite for ML techniques. The min-max scaling normalization technique [37,38] was implemented in this investigation. It converts the data into a specific range of [0, 1].

Equation (1) illustrates the calculation of the min-max scaled value $x^{\prime }$ for a specified parameter *x*:

(1) $$x^{\prime }=\frac{x-\min(x)}{\max(x)-\min(x)}$$

The definitions are as follows:

- *x* is the filtered EEG signal.
- min(x) is the minimum value of the feature.
- max(x) is the maximum value of the feature.

After the data cleansing, correcting, and normalization process, the target variable was subsequently converted into binary categories using the “One-hot encoding” method, with a value of 1 assigned to seizures and 0 assigned to non-seizures. Following that, feature extraction steps were utilized through mathematical transformations, namely the Hurst exponent and DWT, which will be elaborated upon in the subsequent sections. Once these new variables were created, the data underwent normalization. Ultimately, the issue of class imbalance was resolved by reorganizing the data in order to attain a more equitable distribution of cases with seizure and non-seizure instances.

### 3.2. Feature Extraction and Feature Selection

Following the preprocessing stage, the signal undergoes further processing to extract the features of the Hurst exponent and DWT. Initially, the Hurst exponent approach analyzes the signal to identify its characteristics. The Hurst exponent quantifies the extent of long-term dependence in time-series signals [39].

#### 3.2.1. Reasons for Using the Combination of Hurst Exponent Analysis and Discrete Wavelet Transform for Feature Extraction

Conventional feature extraction techniques for EEG characteristics include time-domain feature extraction (e.g., mean amplitude, standard deviation, variance, skewness, kurtosis), power spectral density, fast Fourier transform, short-time Fourier transform, and entropy-based feature extraction, etc. We employ the Hurst exponent analysis in conjunction with DWT for feature extraction due to its ability to identify long-term dependencies and non-stationary characteristics of EEG signals, while Daubechies 4 DWT facilitates multi-resolution analysis, which is advantageous for transient detection.

Table 4 provides a detailed comparison of conventional feature extraction methods with the Hurst exponent and DWT, showcasing their advantages and drawbacks.

#### 3.2.2. Feature Extraction

The intent of this framework is to look into the feasibility of creating a strategy based on the Hurst exponent to pick channel features that are potentially significant for prediction purposes. The proposed hypothesis is that the extended duration of the EEG signals in individuals with epilepsy and no seizure can be used to distinguish between the two groups. To accomplish this goal, we examined the long-term memory features of the EEG using the time-dependent Hurst analysis and the rescaled range (R/S) technique. This entails the division of the time-series EEGs into smaller segments, followed by the calculation of the range and standard deviation for each segment and the subsequent rescaling of the range by the standard deviation.

The Hurst exponent, denoted as *H*, is defined based on the long-term tendency of the *rescaled range* in relation to the time span of a time-series. It is mathematically stated as shown in Equation (2):

(2) $$E\left[\frac{R(n)}{S(n)}\right]=Cn^{H}\;\mathrm{as}\;n\to \infty ,$$

The equation is defined as follows:

- R(n) is the *range* of the first *n* cumulative deviations from the mean;
- S(n) is the series (sum) of the first *n standard deviations*;
- $E\left[x\right]$ is the *expected value*;
- *n* is the time span of the observation (number of data points in a time-series);
- *C* is a constant.

The Hurst exponent (*H*) in computational neuroscience measures the persistence of a time-series EEG signal, indicating the degree to which past values influence future values. This study discovered that both healthy people and those with epilepsy exhibit Hurst exponents demonstrating highly persistent behavior (*H* > 0.5). The brain signal exhibits persistent behavior when the value of *H* exceeds 0.5, suggesting that high values in the signal typically follow high values, and low values typically follow low values. This indicates a pattern of conduct that follows trends over a period of time. The elevated Hurst exponent values also indicate the presence of robust correlation in the data (i.e., the EEG signal has an inclination to persist in its present pattern), resulting in long-term data memory. While the Hurst exponent divergence between healthy and epilepsy participants is minimal, it is significant enough for ML algorithms to discern. Section 5 has a detailed discussion of the outcomes.

Our research utilizes DWT to identify frequency characteristics. Previous understanding of EEG signals indicates that the brain primarily focuses its signal frequency below 30 Hz during motor imaging, with most artifact signals being 5 Hz or lower. Hence, the frequency range that is beneficial in EEG signal data lies within 8–30 Hz. This work utilizes the Daubechies 4 (db4) wavelet to perform a 4th-order wavelet decomposition in order to obtain low-frequency sub-band characteristics. The db4 wavelet is highly efficient in processing EEG signals because of its restricted support and capability to capture the transient and oscillatory characteristics commonly observed in brain activity [40]. Wavelets possess the ability to describe the specific attributes of signals within both the temporal and frequency domains. When employing a smaller scale, the temporal observation range is reduced, but in the frequency domain, it is analogous to utilizing high frequency for high-resolution analysis. In other words, it involves using a high-frequency wavelet for meticulous observation. When using a large scale, the temporal observation range is extensive, and in the frequency domain, it is similar to using a low-frequency wavelet for a broad overview observation.

EEG signals are naturally discrete since they are sampled at particular intervals to measure the electrical contribution of the population of neurons in the brain. Because of its discrete character, the utilization of DWT is especially suitable for studying EEG data. Compared with the continuous wavelet transform, the discrete wavelet transform is to limit the *a* and τ of the wavelet basis function ψ(a,τ) to discrete points, that is, the discretization of scale and displacement, respectively, and the discrete wavelet basis function is shown in Equation (3).

(3) $$\psi_{j,k}\left(t\right)=2^{-\frac{i}{2}}\psi \left(2^{-j}t-k\right),$$

where j∈Z,k∈Z, the DWT is shown in Equation (4):

(4) $$WT_{x}\left(j,k\right)=\int x\left(t\right)\psi_{j,k}^{*}\left(t\right)\;dt.$$

Scaling is necessary for all algorithms that rely on distance measurements. This encompasses many curve-fitting methods, such as linear and nonlinear regressions, logistic regression, KNN, SVM, and neural networks, as well as clustering algorithms like *k*-means clustering.

The features ultimately utilized from the Hurst exponent analysis-based extraction approach are the Hurst exponent ($hurst_{ex}$) and the Hurst constant ($hurst_{c}$). The characteristics ($hurst_{ex}$ and $hurst_{c}$) are further examined using statistical metrics (mean, median) for class 0 (non-seizure) and class 1 (seizure). For each wavelet decomposition coefficient, the following statistical characteristics are calculated: percentile-based features like $n_{5}$ (5th percentile), which denotes the lowest bound of the wavelet coefficients; $n_{25}$ (25th percentile), which denotes the lowest quartile of the wavelet coefficients; $n_{75}$ (75th percentile), which depicts the upper quartile of the wavelet coefficients; $n_{95}$ (95th percentile), which represents the upper boundary of the wavelet coefficients; and basic statistical features like median, mean, standard deviation, variance, and root mean square (RMS). As DWT is a multi-tier decomposition technique, these statistical features are calculated at each level of wavelet decomposition. The feature selection stage will follow this feature extraction strategy using an ANOVA test and a random forest regressor. We will then employ a logistic regression technique to study the importance of features.

#### 3.2.3. Feature Selection

Feature selection is an essential phase in ML that entails identifying the most pertinent features from a dataset while discarding redundant or unnecessary ones. This promotes model efficacy, lowers computing expenses, and improves interpretability.

We conducted a thorough evaluation before feeding inputs into the models to determine whether feature selection enhances accuracy. Initially, we provided all the features to the model. We then used only the selected characteristics as input. Finally, we compared the accuracy and F1-scores. The dataset’s numeric input values and categorical target variable led to the selection of the ANOVA test for feature selection. We also used a random forest regressor to perform feature selection and compared the two methods.

We also examined the importance of the features to comprehend the impact of a change in features on the output of the ML model. In order to evaluate the importance of features, we implemented logistic regression. The subsequent Section 3.3.1 contains additional information regarding the analysis of the features’ importance.

While feature selection can frequently improve accuracy, it did not prove advantageous in this specific instance. Therefore, all features were utilized as input, and an in-depth discussion of the results is discussed in Section 5.

### 3.3. Sensitivity Analysis

In ML, a sensitivity analysis (SA) determines the impact of changing input features, hyperparameters, or model components on the model’s output or accuracy of predictions. This aids in comprehending the dependability, significance of features, and model robustness.

#### 3.3.1. Feature Importance Analysis

Feature importance is a measure of the extent to which the output of a model is influenced by a change in a specific feature.

**Figure 2:**
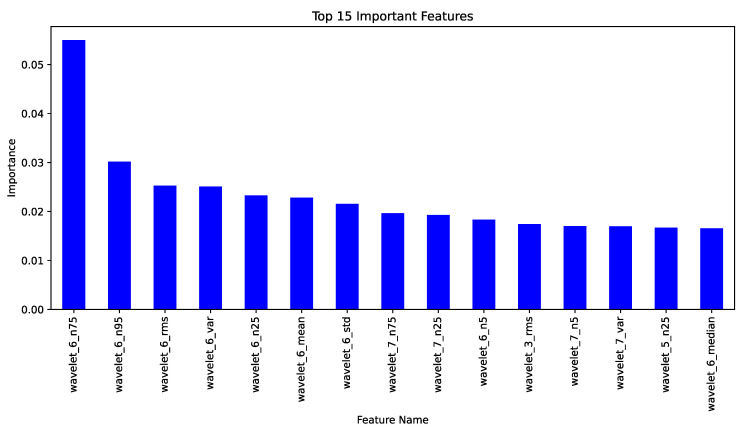
Importance of the top 15 features.

Figure 2 illustrates the 15 most significant characteristics that predominantly influence the classification output. The $n_{75}$ (75th percentile), indicating the upper quartile of the wavelet coefficients, and $n_{95}$ (95th percentile), representing the upper limit of the wavelet coefficients, are identified as the two most significant characteristics. It indicates that they capture moderately high and high amplitudes, respectively, in the wavelet domain, which are frequently associated with epileptic spikes or bursts.

#### 3.3.2. Hyperparameter Sensitivity Analysis

Hyperparameter sensitivity analysis evaluates the impact of varying hyperparameter values on the efficacy of the model. Our research utilizes the grid search approach for hyperparameter optimization. Grid search is a methodical approach for optimizing hyperparameters through a comprehensive examination of a predefined range of values. The chosen hyperparameters for the three machine learning models are outlined in Section 3.4.1, Section 3.4.2, and Section 3.4.3, respectively.

### 3.4. Machine Learning Model Development

The SVM, the RF classifier, and the LSTM network are the three distinct ML models developed and evaluated in this investigation to classify seizures using EEG signals. Each model specifically aims to leverage unique capabilities in processing and analyzing EEG data, providing a comprehensive approach to classification of seizures.

#### 3.4.1. Support Vector Machine (SVM)

The SVM model initially categorizes the dataset using a linear kernel, selecting it for its simplicity and readability. The linear kernel divides data points into distinct classes using a linear decision boundary, a straight line in two dimensions, which is ideal for the properties of our considered dataset.

A dataset containing feature vectors and their corresponding class labels served as the model’s training dataset. In this training phase, the SVM aimed to identify the optimal hyperplane that best separates the classes, enhancing classification accuracy.

**Figure 3:**
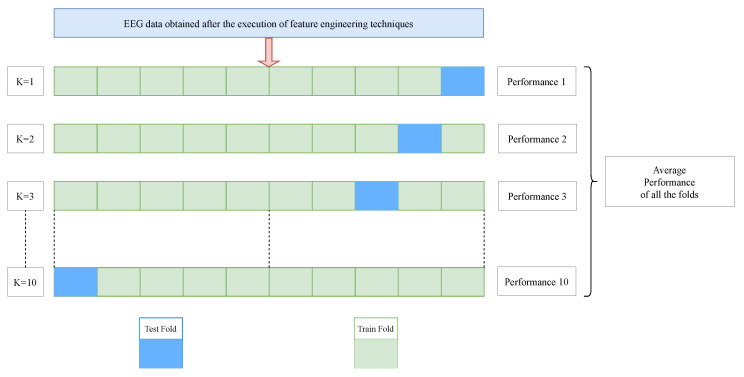
Operation diagram of the 10-fold cross-validation technique.

The generalization capacity of the SVM refers to its ability to accurately classify or predict data that it has not seen before. SVM’s good generalization ability is attributed to factors like its margin maximization, use of support vectors, and use of kernels to handle nonlinear data. In this research paradigm, the generalization capacity of the SVM model is evaluated using a 10-fold cross-validation technique. This resampling technique divides the dataset into 10 groups (or 10 folds/subsets) of identical size, offering a reliable assessment of the model’s performance on fresh data. Nine of these folds are used to train the model, while the remaining one is used for testing. Each fold serves as the test set, guaranteeing that each fold is used as the test set once and this procedure is iterated ten times. The model’s performance metrics such as accuracy, precision, and recall from each test are averaged following all ten iterations. An overall assessment of the model’s performance is given by this average. The operation diagram of 10-fold cross-validation technique is shown in Figure 3. Mathematically, the average performance of all the folds ($Performance_{avg}$) may be represented as shown in Equation (5):

(5) $$Performance_{avg}=\frac{1}{10}\sum_{i=1}^{10}Performance_{i}$$

where $Performance_{i}$ is the individual performance of the ith fold (i=1,2,3,…,10).

This approach proves advantageous for datasets such as the University of Bonn dataset, as it mitigates overfitting by preventing the model from recalling the training data, and generates more consistent performance metrics that accurately reflect the model’s potential performance on unknown data.

The chosen hyperparameters of SVM are [kernel=linear], [probability=True], [class_weight = balanced], and [cv=10].

#### 3.4.2. Random Forest (RF) Classifier

For the categorization of epilepsy, an RF classifier model was used, and its performance was improved by customizing it with a special set of hyperparameters. The settings included a maximum tree depth of 5 to prevent overfitting; a predefined random state to ensure reproducibility of results; and the square root of the total number of features as the number of features assessed for the optimal split. The maximum depth is set at 5 to avoid overfitting [‘*max depth*’: 5], as trees that are overly deep may collect noise in the data instead of patterns. For the increment of the diversity of individual trees and for the improvement of data generalization, the model considers only the square root of the total number of features [‘*maxfeatures*’: ‘*sqrt*’] to find the best split. There were 150 trees in the forest, and it was determined that splitting an internal node requires at least two samples. This setting is a standard and helps prevent splits that are too narrow.

The chosen hyperparameters of RF are [maxdepth:5], [maxfeatures:sqrt], [min_samplesplit: 5], and [n_estimators: 100].

The intrinsic feature of the fitted model was used to evaluate the features’ importance after the classifier had been trained on the training dataset. We chose the top 20 features, ranking the characteristics in decreasing order of significance, to explore this information.

We evaluated the model’s performance using the 10-fold cross-validation method, which we also used in the SVM model. We produced a classification report metric by comparing the actual test labels with the predictions. This report provides a summary of each class’s performance metrics, including accuracy, recall, F1-score, and support. This information is clarified in depth in Table 4 and Table 5, respectively.

#### 3.4.3. The Long Short-Term Memory (LSTM) Network

The LSTM model was created with the intention of accurately capturing long-term dependencies found in the sequential EEG data. This was accomplished by making use of recurrent neural networks’ (RNNs’) capacity for precise sequence prediction. Separate training and testing sets of the dataset were created. After that, the input data were transformed into a three-dimensional array, [samples,timesteps,features] where the dimensions were determined by the number of samples or the number of EEG epochs, time steps or the number of time points in each epoch, and the number of features per time step.

We employed the root mean square propagation (RMSProp) optimizer, recognized for its efficacy in training recurrent neural networks (RNNs) with an adaptive learning rate, and the binary cross-entropy loss function, appropriate for binary classification tasks, to construct the LSTM network. The model’s performance was evaluated using accuracy as the measurement. We trained the network for 50 epochs with a batch size of 72, using training data to enhance the loss function and testing data to assess performance. To enhance the model’s capacity to accurately identify binary outcomes, the training technique maintained the temporal sequence, which is essential for time-dependent data.

The chosen hyperparameters of LSTM are [units=50], [Dropout=(0.1)], [Dense:(1,activation=sigmoid)], [loss = binary_crossentropy], [optimizer=rmsprop], [epochs=50], and [batch_size = 72].

## 4. The Proposed Framework

**Figure 4:**
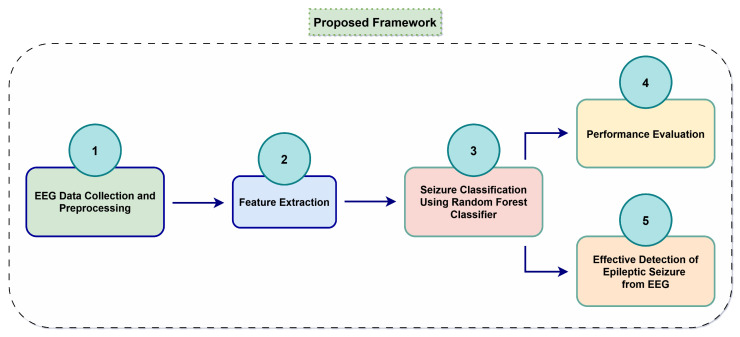
Block diagram representation of the proposed framework.

The comprehensive approach employed in this research is described in Section 3, and the procedural flow is illustrated in Figure 1. Our framework is being proposed in accordance with the necessity and applicability of the individual methodological steps as mentioned in Section 3. Figure 4 presents an easy-to-understand block diagram of the proposed framework, illustrating the basic structure of our research concept for classifying seizures from EEG leveraging ML.

The above figure shows the following details:

- As demonstrated in block 1 of Figure 4, the EEG data collection and essential preprocessing are conducted in accordance with the procedures outlined in the previous Section 3.1.
- The preprocessed EEG data are subsequently transmitted into the feature extraction stage, as illustrated in block 2 of Figure 4. The features are extracted using Hurst exponent analysis and DWT, as previously mentioned in Section 3.2.2. The feature selection stage is not included in this representation because it did not prove to be advantageous in this specific regard, as previously mentioned in Section 3.2.3.
- All retrieved features are subsequently input into the RF classifier for the classification of aberrant epileptic episodes, as seen in block 3 of Figure 4. The selection of the classifier is based exclusively on the superior performance of the RF classifier compared to other models, as seen in Table 5 and Table 6.
- The subsequent phase, as illustrated in block 4 of Figure 4, is to assess the RF classifier’s performance metrics in terms of its ability to classify class 0 (non-seizure) and class 1 (seizure) data.
- The RF classifier’s capability to classify seizures from EEG data has been demonstrated to be satisfactory in Table 5 and Table 6. Consequently, the model may be implemented in other datasets or in real-world medical applications to facilitate effective detection of seizures as depicted in block 5 of Figure 4.

## 5. Results and Discussion

This study focuses on the development and evaluation of three different ML models intended for seizure classification. According to the experiment’s results, the RF classifier has proven to be more effective than the alternative approaches. This experiment uses several performance evaluation matrices, including F1-score, accuracy, precision, and recall.

Table 5 shows the comparison of the three ML models’ performances with proper class separation. Here, class 0 corresponds to non-seizure and class 1 represents seizure. The performance report in Table 5 indicates that the accuracy of both the SVM and RF classifiers is identical (97%); however, the RF classifier demonstrates superior class-wise precision, recall, and F1-scores compared to the SVM classifier, thereby affirming its superiority over the other methods. Notably, the creation of the performance metrics in Table 5 preceded the application of feature selection techniques.

We directly fed the time-series EEG data, incorporating just the preprocessing methods outlined in Section 3.1.2, into the LSTM classifier, and observed the performance metrics shown in Table 5. The LSTM model utilizing all features demonstrates a superior accuracy of 93%, compared to the LSTM model based on time-series EEG, which achieves an accuracy of 92%. The LSTM with features demonstrates superior balanced performance with less complexity.

**Figure 5:**
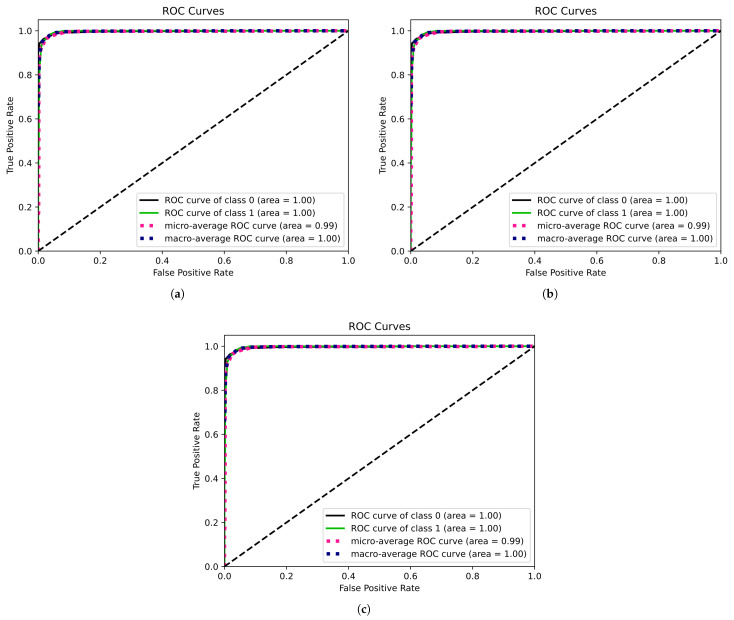
Receiver operating characteristic (ROC) curve for all the ML seizure classification approaches considering all features: (**a**) ROC curve for support vector machine (SVM). (**b**) ROC curve for random forest classifier. (**c**) ROC curve for long short-term memory (LSTM).

Figure 5 displays the receiver operating characteristic (ROC) curves for the various approaches. The RF classifier as shown in Figure 5b exhibited greater performance due to its ensemble learning characteristics, with a higher area under the curve (AUC) indicating enhanced discriminating between the non-seizure and seizure classes. It possesses the capability to recognize true positives, hence enhancing sensitivity, which is a critical component in medical diagnostics. However, other approaches have shown significant efficacy in class separation, as evidenced by the ROC curves.

**Figure 6:**
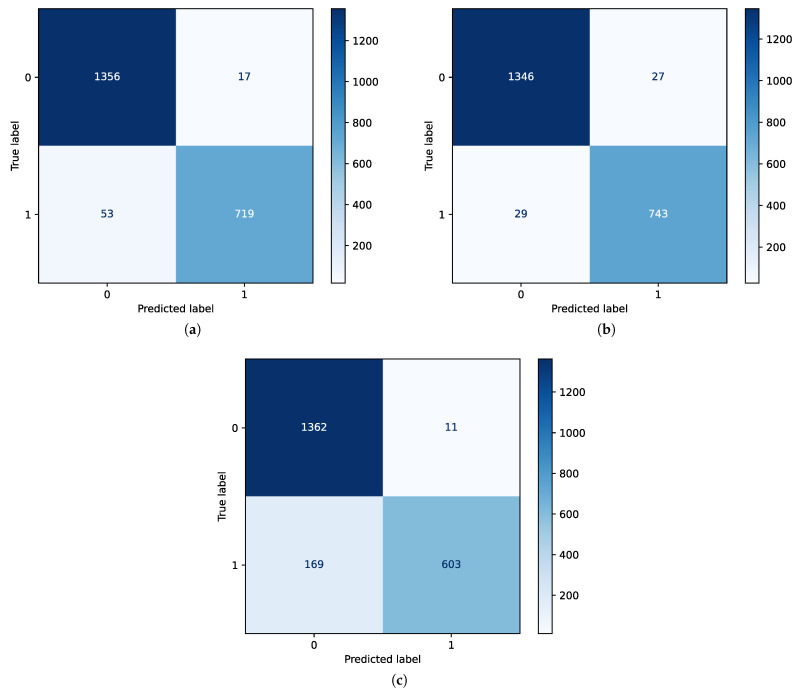
Confusion matrix (CFM) showing the performance of the respective ML models considering all features: (**a**) CFM for SVM classifier with all features. (**b**) CFM for RF classifier with all features. (**c**) CFM for LSTM classifier with all features.

**Figure 7:**
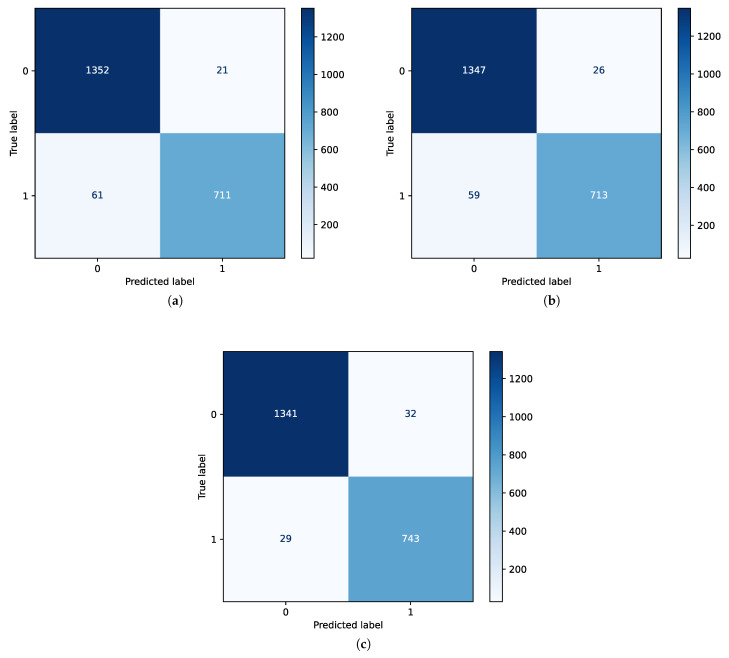
Confusion matrix (CFM) showing the performance of the respective ML models considering selected features: (**a**) CFM for SVM classifier with only the ANOVA test’s features. (**b**) CFM for SVM classifier considering the features of random forest regression technique. (**c**) CFM for RF classifier with ANOVA features.

The confusion matrices presented in Figure 6 demonstrate the performance of the three ML techniques with the inclusion of all features. Here, class 0 corresponds to non-seizure and class 1 represents seizure. The confusion matrices indicate that the RF classifier, as shown in Figure 6b, has superior performance among the methods. Figure 7 demonstrates the performance of SVM and RF classifiers after the application of feature selection with an ANOVA test and random forest regression techniques.

In addition, Table 6 displays the outcomes of the different methods after conducting feature selection. We initially extracted the top 20 features using the ANOVA method. Later, we employed the random forest feature selection technique to extract another set of the top 20 features. Table 6 shows the performance of SVM and RF classifiers after the application of the feature selection techniques, as discussed in Section 3.2.3. The application of feature engineering can be avoided for time-series signals using LSTM, as they follow the recurrent neural network (RNN)/autoencoder approach and therefore are not included in Table 6.

Despite the potential benefits of feature selection in enhancing accuracy in various scenarios, its effectiveness was not demonstrated in this particular example. Therefore, we decided to use all data as input and select RF as the optimal classification model based on performance metrics.

**Figure 8:**
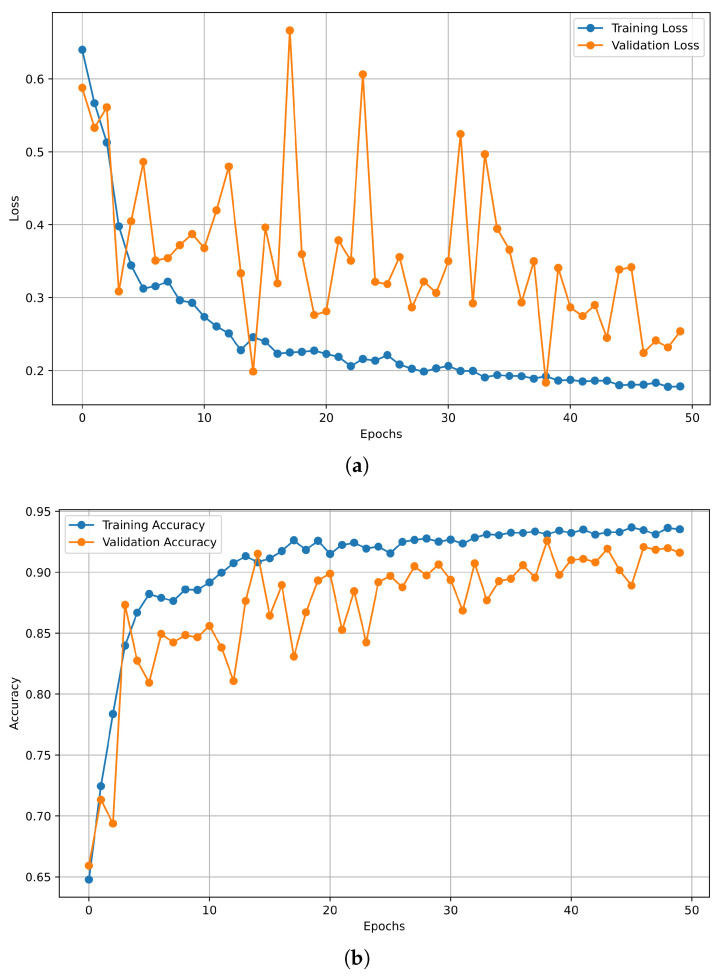
Plot related to LSTM classifier with loss vs. epochs and accuracy vs. epochs curves: (**a**) Plot of LSTM model loss over epochs. (**b**) Plot of LSTM model accuracy over epochs.

Figure 8a,b illustrate the loss and model accuracy over epochs, respectively, reflecting the performance of the LSTM classification model. These two graphs are essential for comprehending the training process, recognizing possible difficulties, and assessing the model’s performance.

The subsequent parameters were taken into account for the visualization of loss and accuracy curves over epochs for the LSTM classifier, respectively:

1. Axes:
  - X-Axis: Denotes the epoch count, ranging from 1 to 50.
  - Y-Axis: Represents the loss in Figure 8a and denotes the accuracy of the model in Figure 8b, respectively.
2. Lines:
  - Training Loss: Indicates the model’s performance on the training dataset.
  - Validation Loss: Reflects the model’s efficacy on previously unseen validation data.
  - Training Accuracy: Demonstrates the model’s efficacy in classifying the training data.
  - Validation Accuracy: Indicates the model’s ability to generalize to previously unseen validation data.
3. Number of epochs: 50.
4. Batch size: 72.

Figure 8a illustrates a pronounced decline in training loss, signifying that the model learns patterns from the training dataset while exhibiting a variable trend in validation loss. The training accuracy plot in Figure 8b demonstrates good performance; however, the validation accuracy line indicates a lack of generalization to unseen validation data.

Upon evaluating the individual performances of the three models in differentiating between non-seizure and seizure data from EEG signals, we concluded that the RF classifier is the best-performing classifier that we have developed in this framework and surpassed the other two models when every feature was utilized as input.

### 5.1. Confidence Interval Analysis

When classifying epilepsy based on EEG, a confidence interval (CI) shows the range of values that the actual classification performance (like accuracy, sensitivity, and specificity) is likely to fall within [41]. A 95% confidence interval (95% CI), which is a standard in medical applications, indicates that if the classification procedure were conducted repeatedly on various EEG samples, 95% of the resulting intervals would include the genuine classification performance. This quantifies the uncertainty in model performance resulting from data fluctuations.

We then utilize resampling with replacement to create bootstrap samples, each including the same number of observations as the original dataset [42]. Our research employs 1000 bootstrap samples. The subsequent step involves calculating the percentile-based confidence interval as follows:

[Lowerbound = Percentile at 2.5%] and [Upperbound = Percentile at 97.5%]

Mathematically, CI can be calculated as shown in Equation (6):

(6) CI=μ±1.96×σ

The equation is defined as follows:

- μ is the mean of bootstrap metric values.
- σ is the standard deviation of bootstrap metric values.

Table 7 presents a comprehensive comparison of the confidence intervals for several performance metrics (precision, recall, and F1-score) among the three classification methods. The narrow confidence range (0.0066 for accuracy, 0.0146 for precision, 0.0145 for recall, and 0.0145 for the F1-score) of the RF classifier, including all features, denotes low variation, indicating that the model’s performance remains stable across several samples.

Cohen’s formula [43,44] was employed to calculate the effect size as shown in Equations (7) and (8).

(7) $$d=\frac{{\bar{x}}_{1}-{\bar{x}}_{2}}{S_{\mathrm{pooled}}}$$

where *d* is Cohen’s d effect size, $S_{pooled}$ is the pooled standard deviation, and ${\bar{x}}_{1}$ and ${\bar{x}}_{2}$ are the values derived from class 0 and class 1.

(8) $$S_{\mathrm{pooled}}=\sqrt{\frac{\left(n_{1}-1\right)sd_{1}^{2}+\left(n_{2}-1\right)sd_{2}^{2}}{ss_{1}+ss_{2}-2}}$$

where $sd_{1}$ and $sd_{2}$ are the class-specific (seizure and non-seizure) standard deviation, $ss_{1}$ and $ss_{2}$ are the class-specific numbers of samples. $n_{1}$ and $n_{2}$ are the number of observations in two classes.

We have determined Cohen’s d for the three models by employing Equations (7) and (8). The Cohen’s d for RF is 0.8, which is the most favorable of the three methods. This value suggests a very large effect size, indicating a significant distinction between the two categories seizure and non-seizure when classified by RF. This measurement proves the efficacy of the RF classifier.

### 5.2. Computational Efficiency Analysis

Table 8 presents a comprehensive evaluation of the computational efficiency of several machine learning categorization methods. The table clearly indicates that the deep learning-based LSTM model requires the most execution time in comparison to the SVM and RF models. The most suitable model from our research framework, RF, requires 6.7665 s for training and inference execution. The execution time of the SVM model is the least among all classification methods; nevertheless, its performance is lower than that of RF when utilizing every feature as input.

### 5.3. Performance Comparison of the Proposed Method with Existing Frameworks

The proposed framework has been evaluated against the existing framework based on multiple performance indicators such as accuracy, sensitivity, specificity and false positive rate (FPR). The entire analysis can be found in Table 9. We find that the proposed framework functions very well and is more trustworthy than previous frameworks with a remarkable accuracy of 97%, sensitivity of 97.20%, specificity of 97.30%, and a false positive rate as low as 0.0271.

The following are limitations of the existing works with which the proposed framework is compared:

- The research by Selvekumari et al. [45] and Zabihi et al. [46] is limited by the fact that it is patient-specific. This makes it harder to use in real life. The approach by Selvakumari et al. [45] entails high-dimensional phase space reconstruction, rendering it computationally expensive. Zabihi et al. [46] also did not check its generalizability on other benchmark epileptic EEG databases.
- In order to rectify the disparity between pre-ictal and interictal data, the research conducted by Zhang et al. [47] condenses segmented pre-ictal signals to produce artificial pre-ictal signals. While this method mitigates data imbalance, it may introduce synthetic patterns that are not present in natural EEG signals, which potentially impacts the model’s performance on actual pre-ictal data.
- The work of Jana et al. [48] is limited to only one dataset. Since they have not employed frequency-level feature extraction, their accuracy is not up to the mark because of the presence of common artifacts caused by eye blinking and muscle activities during EEG recordings. They also used a 1 s EEG sample from multi-channel EEG to train their dense convolution neural network.

The enhanced accuracy of the proposed technique results from its efficient feature extraction strategies that capture non-stationary EEG characteristics through Hurst exponent analysis, facilitate multi-level decomposition via DWT, and utilize finely tuned hyperparameters of the RF classifier.

The following inferences may be made by monitoring the performance metrics of the proposed framework:

- The RF classifier guarantees reliable classification of both seizure and non-seizure conditions, achieving an accuracy of 97%. This degree of accuracy may enhance patient outcomes by enabling early and correct interventions in epileptic conditions, while also optimizing the utilization of medical assets.
- The high sensitivity of this model (97.20%) has the potential to significantly impact medical diagnostics by classifying non-seizure and seizure conditions in EEG biosignals. Its capacity to reduce false negatives enables its application in the early diagnosis of epileptic disorders.
- The high specificity of 97.30% indicates the model’s proficiency in accurately recognizing negative cases (i.e., non-seizure instances).
- A false positive rate as low as 0.0271 is a highly sought-after key parameter in medical diagnostics, since maintaining it at a low level is vital for assuring accuracy and dependability in healthcare applications.

### 5.4. Assessment of the Generalizability of the Proposed Framework for Alternative Datasets

To guarantee the model’s dependability on new, unseen data instead of simply the dataset it was trained on, generalizability is essential for medical classification models, particularly in EEG-based epilepsy detection. In order to evaluate the generalizability of our proposed framework, which incorporates all features as input, we have utilized the CHB-MIT scalp EEG database [49,50], which is a compilation of EEG recordings from 22 pediatric subjects with intractable seizures.

In order to preserve the single-channel character of the University of Bonn dataset, we have chosen the single-channel EEG from CZ-PZ (which is the principal channel for epilepsy-related brain activation [51]) in the CHB-MIT scalp EEG database. Subsequently, the data underwent preprocessing phases, as described in Section 3.1.2, and feature extraction and selection as described in Section 3.2. The information is then fed to our proposed framework of RF, which serves as the classifier, and all features are taken into account.

Utilizing our suggested classification framework RF on the CHB-MIT scalp EEG database, we achieved the following results as shown in Table 10:

Here, in Table 10, class 0 corresponds to non-seizure and class 1 represents seizure. The data in Table 10 clearly demonstrate that the proposed technique effectively classifies seizures from EEG in previously unobserved data, hence establishing its reliability due to its remarkable generalizability on new/unseen data.

## 6. Conclusions

In summary, the use of ML methods to identify epilepsy using EEG data represents a significant advancement in medical diagnosis. EEG offers a substantial amount of data that ML algorithms can efficiently examine to identify aberrant brain activity that is symptomatic of epilepsy. ML models, including SVM, RF classifiers, and LSTM, have demonstrated high accuracy and reliability in identifying epileptic seizures, offering promising outcomes. The experiment’s findings demonstrate that the RF classifier is more promising than the other alternatives, with an accuracy of 97% and a sensitivity of 97.20%. From the performance comparison report shown in Table 9, it can be stated that the approach yielded satisfactory results as compared to the existing research works. It is also worth mentioning that the proposed framework performs better without the employment of feature selection. Table 8 demonstrates that the suggested framework is computationally efficient regarding execution time, rendering it advantageous for implementation on edge hardware. The developed research framework has a strong capacity for generalizing unobserved data, as demonstrated in Table 10, where it displayed exceptional generalizability on the CHB-MIT scalp EEG database. The robust generalizability of our study renders it a dependable option for practical medical applications.

### 6.1. Limitations and Practical Implementation Challenges

The following are the limitations and challenges within this work:

- In conjunction with DWT, the feature extraction stage implements Hurst exponent analysis. Although the Hurst exponent is capable of detecting non-stationarity of EEG and capturing long-term dependencies, it may require the inclusion of additional features to distinguish between the two classes, as it is unable to discriminate between the classes independently.
- Although the RF classifier utilizing all features demonstrates superior performance among the three machine learning methodologies, for hardware implementation, we may opt for the RF model incorporating only the ANOVA features, as it exhibits an execution time of 0.5688 s, peak memory consumption of 1.81 MB, and a single sample inference duration of 0.003044 s as shown in Table 8. To mitigate practical implementation challenges, we must embrace this configuration, since it will yield minimal computing costs for real-world medical applications.
- This developed research approach has been validated with two publicly accessible benchmark EEG datasets, specifically the University of Bonn dataset and the CHB-MIT scalp EEG database, all of which are annotated by medical professionals. Nonetheless, the established framework has not undergone post-deployment medical validation using real-time patient epileptic EEG data. Consequently, the evaluation of generalizability in practical clinical applications is not reported.

### 6.2. Future Research Direction

The following are the future research directions of this work:

- Initiatives will be undertaken to deploy the established framework on edge hardware for real-time inference. The use of edge hardware will facilitate the streamlining of real-time validation for our clinical testing system.
- This framework will facilitate the early detection and has the potential to develop personalized treatment strategies for epileptic seizures that are specific to individual EEG patterns.

## Figures and Tables

**Table 1:** Summary of the related literature.

Reference	Dataset Used	Methods of Classification	Inference	Shortcomings
Shah et al. [26]	CHB-MIT and University of BONN	Random neural network (RNN)	Accuracies of 93.27% and 99.84%, on CHB-MIT and BONN dataset, respectively.	The study amalgamates multi-channel (CHB-MIT) with single-channel (University of Bonn) EEG data from many patients, potentially resulting in information leakage between the training and testing datasets.
Kunekar et al. [27]	UCI-Epileptic Seizure Dataset	Logistic regression, K-nearest neighbors (KNN), SVM, artificial neural networks (ANNs), and LSTM	SVM classifier attained a validation accuracy of 97.2.%	Computationally expensive, which may restrict their use in limited-resources contexts.
Yang et al. [28]	Children’s Hospital at Zhejiang University School of Medicine	Error-correcting output codes (ECOC) SVM, decision tree, KNN, and RF	Accuracy of SVM is 98.15% with EEG+ECG as inputs.	Limited sample size; no assessment of the method’s computing efficiency.
Yogarajan et al. [29]	University of BONN	Deep neural network (DNN) and binary dragonfly algorithm (BDFA)	Accuracy of 100% with 13% selected feature subset.	Computationally expensive; the dataset size and diversity are not reported.
Tuncer et al. [30]	Temple University Hospital EEG dataset	KNN, SVM, RF, and LSTM	Accuracy of 95.92% for the four-class case and 98.08% for the two-class case using LSTM.	Computationally expensive; restricted dataset diversity.
Mardini et al. [31]	University of BONN	SVM, KNN, ANN, and Naive Bayes	Average accuracy of 97.82% with ANN.	Computationally demanding; overfitting may lead to decreased efficiency when applied to unobserved data.
Orosco et al. [32]	CHB-MIT scalp EEG database	Linear discriminant analysis (LDA) and pattern recognition neural network (PRNN)	Mean sensitivity of 92.6% with LDA detector.	In the absence of patient-specific calibration, the algorithm may erroneously classify normal EEG as seizures, resulting in an increased incidence of false positives.

**Table 2:** Description of the University of Bonn dataset with collection phase and structure.

Datasets	Subjects	Acquisition Setup	Data Collection Phase
A	Healthy	Surface EEG	Eyes Open (EO)
B	Healthy	Surface EEG	Eyes Closed (EC)
C	Epilepsy patient	Intracranial EEG	Interictal
D	Epilepsy patient	Intracranial EEG	Interictal
E	Epilepsy patient	Intracranial EEG	Seizure

**Table 3:** Dataset attributes of the University of Bonn dataset.

Attributes	Character/Value
Dataset Characteristics	Multivariate, Time-Series
Number of Instances	11,500
Attribute Characteristics	Integer, Real
Number of Attributes	179

**Table 4:** Comparison of conventional feature extraction methods with Hurst exponent and DWT.

Feature Extraction Technique	Advantages	Drawbacks
Time-Domain Features	Low computational complexity	Unable to capture long-term EEG dependencies, and non-stationarity of EEG
Power Spectral Density (PSD)	Captures and records frequency content	Loses temporal information and assumes stationarity
Fast Fourier Transform (FFT)	Effective for periodic signals	Unsuitable for brief occurrences such as epileptic episodes
Short-Time Fourier Transform (STFT)	Depicts time–frequency components	Restricted resolution due to fixed window size
Entropy-Based Features	Captures signal complexity	Computationally expensive
Hurst Exponent	Captures long-term dependencies and non-stationary characteristics of EEG	Possible need for additional features in order to differentiate
Daubechies 4 Discrete Wavelet Transform (DWT)	Multi-resolution analysis is useful for detecting transients; offers compact support and smoothness	Requires expertise to interpret the wavelet coefficients

**Table 5:** Performance comparisons of the various ML classification approaches without feature selection.

		Precision		Recall		F1-Score	
Modalities	Accuracy	Class 0	Class 1	Class 0	Class 1	Class 0	Class 1
SVM	97%	96%	98%	99%	93%	97%	95%
Random Forest	97%	98%	96%	98%	96%	98%	96%
LSTM	93%	92%	93%	88%	85%	90%	91%
LSTM (Time-series EEG)	92%	89%	98%	99%	78%	94%	87%

**Table 6:** Performance comparisons of the various ML classification approaches after employing feature selection.

		Precision		Recall		F1-Score	
Modalities	Accuracy	Class 0	Class 1	Class 0	Class 1	Class 0	Class 1
SVM (ANOVA)	96%	96%	97%	98%	92%	97%	95%
SVM (Random Forest)	96%	96%	96%	98%	92%	97%	94%
RF (ANOVA)	96.5%	98%	96%	98%	96%	98%	96%

**Table 7:** Exhaustive comparison of confidence intervals of various performance measures of the ML classification approaches.

Model	95% Confidence Interval for Accuracy	95% Confidence Interval for Precision	95% Confidence Interval for Recall	95% Confidence Interval for F1-Score
SVM (All Features Included)	(0.9594, 0.9744)	(0.9539, 0.9699)	(0.9538, 0.9697)	(0.9535, 0.9696)
SVM (Only ANOVA Features Included)	(0.9580, 0.9641)	(0.9516, 0.9689)	(0.9515, 0.9688)	(0.9512, 0.9687)
RF (All Features Included)	(0.9667, 0.9733)	(0.9622, 0.9768)	(0.9622, 0.9767)	(0.9622, 0.9767)
RF (Only ANOVA Features Included)	(0.9637, 0.9711)	(0.9339, 0.9526)	(0.9338, 0.9524)	(0.9332, 0.9521)
LSTM (With Features)	(0.9250, 0.9350)	(0.9064, 0.9279)	(0.8993, 0.9235)	(0.8963, 0.9216)
LSTM (Without Features)	(0.9126, 0.9234)	(0.8963, 0.9216)	(0.7597, 0.8150)	(0.8525, 0.8892)

**Table 8:** Exhaustive comparison of computational efficiency of various ML classification approaches.

Model	Execution Time	Peak Memory Usage	Training Time	Single Sample Inference Time
SVM (All Features Included)	0.4308 s	344.8750 MB	0.4308 s	0.000823 s
SVM (Only ANOVA Features Included)	0.3517 s	348.2500 MB	0.3517 s	0.000283 s
RF (All Features Included)	6.7665 s	380.1328 MB	6.7665 s	0.002850 s
RF (Only ANOVA Features Included)	0.5688 s	1.81 MB	0.0430 s	0.003044 s
LSTM (With Features)	34.4397 s	37.62 MB	33.3744 s	0.051009 s
LSTM (Without Features)	38.6160 s	5.15 MB	33.9226 s	0.048969 s

**Table 9:** Performance comparison of the proposed method with existing frameworks.

Methods	Accuracy	Sensitivity	Specificity/FPR
Selvakumari et al. [45]	96.28%	97.5%	94.5%
Zabihi et al. [46]	94.69%	89.1%	94.8%
Zhang et al. [47]	90%	92.2%	FPR = 0.1200
Jana et al. [48]	90.66%	97%	95.87%, FPR = 0.0413
This Work	97%	97.20%	97.30%, FPR = 0.0271

**Table 10:** Performance metrics of the RF (with all features) classification approach on the CHB-MIT scalp EEG database.

		Precision		Recall		F1-Score	
Modality	Accuracy	Class 0	Class 1	Class 0	Class 1	Class 0	Class 1
Random Forest	92%	93%	90%	90%	93%	92%	92%

## Data Availability

The original data presented and utilized in the study are openly available at https://www.ukbonn.de/epileptologie/arbeitsgruppen/ag-lehnertz-neurophysik/downloads/ (University of Bonn dataset) (accessed on 30 October 2024) and https://physionet.org/content/chbmit/1.0.0/ (CHB-MIT scalp EEG database) (15 February 2025). The detailed discussion about these datasets can be found in [35,49,50], respectively.
